# Administration of adipose-derived stem cells extracellular vesicles in a murine model of spinal muscular atrophy: effects of a new potential therapeutic strategy

**DOI:** 10.1186/s13287-024-03693-5

**Published:** 2024-04-01

**Authors:** Federica Virla, Ermanna Turano, Ilaria Scambi, Lorenzo Schiaffino, Marina Boido, Raffaella Mariotti

**Affiliations:** 1 Department of Neuroscience, Biomedicine and Movement Sciences, University of Verona, Verona, Italy; 2 Neuroscience Institute Cavalieri Ottolenghi, Department of Neuroscience “Rita Levi Montalcini”, University of Turin, Turin, Italy

**Keywords:** Extracellular vesicles, Stem cells, Adipose mesenchymal stem cells, SMA, SMNΔ7, Motor neuron disease

## Abstract

**Background:**

Spinal Muscular Atrophy (SMA) is an autosomal-recessive neuromuscular disease affecting children. It is caused by the mutation or deletion of the *survival motor neuron 1* (*SMN1*) gene resulting in lower motor neuron (MN) degeneration followed by motor impairment, progressive skeletal muscle paralysis and respiratory failure. In addition to the already existing therapies, a possible combinatorial strategy could be represented by the use of adipose-derived mesenchymal stem cells (ASCs) that can be obtained easily and in large amounts from adipose tissue. Their efficacy seems to be correlated to their paracrine activity and the production of soluble factors released through extracellular vesicles (EVs). EVs are important mediators of intercellular communication with a diameter between 30 and 100 nm. Their use in other neurodegenerative disorders showed a neuroprotective effect thanks to the release of their content, especially proteins, miRNAs and mRNAs.

**Methods:**

In this study, we evaluated the effect of EVs isolated from ASCs (ASC-EVs) in the SMNΔ7 mice, a severe SMA model. With this purpose, we performed two administrations of ASC-EVs (0.5 µg) in SMA pups via intracerebroventricular injections at post-natal day 3 (P3) and P6. We then assessed the treatment efficacy by behavioural test from P2 to P10 and histological analyses at P10.

**Results:**

The results showed positive effects of ASC-EVs on the disease progression, with improved motor performance and a significant delay in spinal MN degeneration of treated animals. ASC-EVs could also reduce the apoptotic activation (cleaved Caspase-3) and modulate the neuroinflammation with an observed decreased glial activation in lumbar spinal cord, while at peripheral level ASC-EVs could only partially limit the muscular atrophy and fiber denervation.

**Conclusions:**

Our results could encourage the use of ASC-EVs as a therapeutic combinatorial treatment for SMA, bypassing the controversial use of stem cells.

**Supplementary information:**

The online version contains supplementary material available at 10.1186/s13287-024-03693-5.

## Background

Spinal Muscular Atrophy (SMA) is an autosomal recessive neuromuscular disease, with estimated prevalence of 1–2 per 100,000 persons and incidence around 1 in 10,000 live births for SMA type I, which accounts for around 60% of all cases [1]. It is characterized by the selective degeneration and loss of lower motor neurons (MNs). The typical symptoms consist in hypotonia, muscular atrophy and weakness due to the denervation, till the final paralysis of the muscles of both upper and lower limbs as well as of the trunk [2, 3].

Depending on the age of symptom onset and on the achieved motor milestones, five main phenotypes have been described, from the more severe type 0 SMA to the milder type IV [4]. Despite the great variability of phenotypic expression, SMA is due to well-known genetic causes; in particular, homozygous deletions or mutations of the *survival motor neuron 1* (*SMN1*) telomeric gene, encoding for the functional full length SMN (FL-SMN) protein [5]. Humans also display a centromeric copy of the gene, *SMN2*; it differs from the *SMN1* copy for a C to T transition in exon 7 that results in splicing defects and that in most cases leads to the production of a truncated, unstable and not functional form of the protein, called SMNΔ7 [6]. The number of copies of the *SMN2* gene are critical for determining the phenotype and the severity of the disease. Indeed, *SMN2* is able to produce the FL-SMN protein in a low percentage, but not fully sufficiently to compensate the *SMN1* loss [7].

Currently, three SMN-dependent treatments have been approved by FDA and EMA: the antisense oligonucleotide Nusinersen (Spinraza, Biogen) and the small molecule Risdiplam (Evrysdi, Roche) are both splicing modifiers and act to boost the production of FL-SMN protein from *SMN2* gene. The third one is Onasemnogene abeparvovec (Zolgensma, Novartis) and uses the viral vectors strategy to deliver a functional copy of the *SMN1* gene to SMA patients (reviewed in [8]).

Despite the important contribution of these approved drugs in the treatment of SMA, some limitations and concerns still remain, such as patient inclusion criteria, the still unknown long-term effects, the possible treatment-related toxicity, and lastly the high cost of these therapies. Moreover, although SMA is widely considered a MN disease, it has been confirmed that reduced levels of SMN can also affect different cells and tissues, other than motor neurons: in the first case, early dysfunction of sensory neurons and disruption of sensory-motor circuits has been widely demonstrated in preclinical studies [9]. In the second case, multi-organ involvement in SMA leads to a variety of symptoms, ranging from vascular and cardiac alterations, to immune system abnormalities and peripheral nerve involvement [10–12]. It also emerged that SMA is likely a non-cell autonomous disease in which several signalling pathways and molecular/cellular mechanisms are involved, such as the activation of glial cells that could precede, sustain and spread the degenerating process [13]. Also autophagy dysregulation [14–16] and dysregulated cellular signalling, including for example Rho kinase (ROCK) and the extracellular-regulated kinase (ERK) pathways, can act as potential mechanisms of MN degeneration in SMA [17]. Thus, the implementation and further development of SMN-independent therapies appears to be extremely relevant to enhance the beneficial effect of SMN-dependent strategies [4], such as the ones already approved or others in pre-clinical studies [18, 19].

In this sense, a promising therapeutic option in neurodegenerative diseases is represented by mesenchymal stem cells (MSCs): indeed, they can be easily isolated from a variety of different tissues, they are able to migrate to damage tissues stimulating reparative and regenerative processes [20–22], as demonstrated in different disease models, such as spinal cord injury [23], experimental autoimmune encephalomyelitis [24] and amyotrophic lateral sclerosis (ALS) [25–27] murine models. Among MSCs, adipose-derived stem cells (ASCs) are accessible in large amount and could be easily used in autologous transplantation [28].

However, despite many advantages of MSCs-based therapy, various challenges limit their clinical application: for example, the engraftment and differentiation of MSCs in the central nervous system (CNS) tissues, after their transplantation, result in a small percentage. Moreover, given the high proliferative capacity of stem cells, the risk of oncogenic transformation and their immune-rejection should be avoided. Given that, it would be crucial to identify the correct route of administration, the dose of cells and the number of cell-injections to deliver MSCs to the CNS [29].

It is now well accepted that MSCs act through paracrine mechanisms by releasing their content in extracellular vesicles (EVs) [30, 31]. EVs are considered important mediators in intercellular communication as they can transfer their cargo, including proteins, miRNAs and mRNAs to nearby cells in several physiological and pathological conditions [32]. Furthermore, EVs maintain the neuroprotective role of their parental MSCs, as demonstrated by several groups [33, 34]. Indeed, MSCs-derived EVs promote immunomodulation and anti-inflammatory signalling [35, 36], inhibit apoptosis [37] and promote neural plasticity and neurogenesis [38, 39].

Given the remarkable results obtained by our previous studies in in vitro and in vivo ALS models [40, 41] (another MN disease), in the present study we evaluated whether ASCs-derived EVs (ASC-EVs) could exert their neuroprotective role also in SMNΔ7 mice, a largely used model of severe SMA. Here we demonstrated that intracerebroventricular (ICV) injections of ASC-EVs could delay lumbar MN degeneration, and in turn, partly limit the neuromuscular junction (NMJ) degeneration/denervation and skeletal muscle atrophy; we also observed a decrease in the glial activation in SMNΔ7 treated animals, compared to vehicle-treated mice. By inducing such neuroprotective and anti-inflammatory effects, the ASC-EVs administration was also able to improve the motor performance of SMA pups.

Approved SMN-dependent drugs showed an impressive but yet limited effect. Therefore, complementary SMN-independent strategies could be needed to address irreversible degenerative processes [8, 42, 43]. Taken together, our results demonstrate the neuroprotective contribution of this non-cellular and SMN-independent strategy and pave the way for a synergic therapy in combination with SMN-dependent drugs, as proposed in different studies [44, 45] and for different neurodegenerative disorders [46].

## Methods

### ASCs culture

Murine ASCs were isolated from inguinal adipose tissues of 8–12 week-old C57Bl6/J mice (*n* = 5) (Charles River Laboratories, Sant’Angelo Lodigiano, Italy) sacrificed by cervical dislocation. Animals were housed in pathogen-free, climate-controlled facilities and were provided with food and water ad libitum according to current European Community laws. All mouse experiments were carried out in accordance with experimental guidelines approved by the University of Verona committee on animal research (Centro Interdipartimentale di Servizio alla Ricerca Sperimentale) and by the Italian Ministry of Health (protocol #642/2021-PR, title “New therapeutical approach for ALS”). Animal care and all experimental procedures were conducted following the Animal Research: Reporting of In Vivo Experiments (ARRIVE) guidelines. The research complies with the commonly accepted “3Rs”, minimizing the number of animals used and avoiding their suffering.

The isolation of stromal vascular fraction was carried out as previously described [47]. Briefly, the extracellular matrix was incubated in Hank’s Balanced Salt Solution (Life Technologies Italia, Milan, Italy) with collagenase type I (Life Technologies Italia, Milan, Italy) and bovine serum albumin (BSA, AppliChem Nova Chimica Srl, Milan, Italy), centrifuged, and suspended in NH_4_Cl. The fraction was centrifuged again and filtered through a 40 μm nylon mesh to remove cell debris. ASCs were cultured using DMEM, 10% FBS, 100 U/mL penicillin, and 100 µg/mL streptomycin (all from GIBCO Life Technologies, Milan, Italy) and incubated at 37 °C/5% CO_2_.

The immunophenotypic analysis of murine ASCs was performed using monoclonal antibodies specific for CD106, CD29, CD44, CD80, and CD138 and by the absence of hematopoietic and endothelial markers (as CD45, CD11c and CD31), as previously described [48, 49].

### ASCs extracellular vesicles isolation and characterization

ASC-EVs were isolated from the culture medium of 1 × 10^7^ murine ASCs at 14–18 passages. The cells were cultured to confluence and 48 h of FBS deprivation was made to avoid any contamination of vesicles from serum. Then, cell culture supernatant was collected and EVs were obtained using Pure Exo Exosomes Isolation Kit (101Bio, Montain View, CA, USA), following the manufacturer’s protocol. The protein content of EVs was determined by Bicinchoninic Protein Assay (BCA method) using the manufacturer’s protocol (Thermo Scientific™, Milan, Italy BCA™ Protein Assay). ASC-EVs were pooled and used fresh for in vivo administration.

To characterize ASC-EVs, their size distribution and concentration were measured by Nanoparticle Tracking Analysis (NTA) using a Nanosight NS300 (Malvern Instruments, UK). For the measurements, five video recordings with a duration of 1 min were carried out for each sample. Camera level and the detection threshold were set in the acquisition and analysis, respectively, in order to achieve a concentration between 20 and 120 particles/frame. The NTA 3.4 software version was used to acquire and analyse the sample videos. The results are reported as the mean ± SEM of 5 measurements. For size determination, the data are reported as the statistical mode ± SEM of 5 measurements.

To perform electron microscopy ASC-EVs pellets were fixed in 2% glutaraldehyde in Sorensen buffer (pH 7.4) for 2 h, and then post-fixed in 1% osmium tetroxide (OsO_4_) in aqueous solution for 2 h. The sample was dehydrated in graded concentrations of acetone and embedded in Epon-Araldite mixture (Electron Microscopy Sciences, Fort Washington, PA, USA). The semithin Sect. (1 μm in thickness) were examined by light microscopy (Olympus BX51, Olympus Optical, Hamburg, Germany) and stained with toluidine blue. The ultrathin sections were cut at a 70 nm thickness, placed on Cu/Rh grids with Ultracut E (Reichert,Wien, Austria), and observed with transmission electron microscopy (TEM) using a Morgagni 268D electron microscope (Philips).

To confirm ASC-EVs isolation and purity the immunoblotting analysis was performed: ASC-EVs proteins were denatured, separated on 4–12% polyacrylamide gels and transferred onto a nitrocellulose membrane. Antibodies against murine HSP70 (70 kDa, 1:100 HSP70 (K-20): sc-1060 Santa Cruz Biotechnology, DBA Italia Srl, Milan, Italy) and CD9 (25 kDa, 1:100 MM2/57, Millipore CBL-162) were used. After incubation with IgG HRP-conjugated secondary antibodies (Dako Agilent, Milan, Italy) the membranes were incubated with a chemiluminescent HRP substrate and detected with G:BOX F3 GeneSys (Syngene, Cambridge, UK). ASCs lysates were used as a positive control.

### SMA animals

SMN2+/+; SMNΔ7+/+; SMN+/- mice (stock number 005025; Jackson Lab, Bar Harbor, ME, USA) were bred to obtain the experimental animals SMN-/- (SMA, as model of severe SMA) and SMN+/+ (WT) offspring. SMA and WT pups were left in the cage with the mother until the sacrifice at post-natal day 10 (P10). Pups of both sexes were used in this study. Animals had free access to food and water, and were kept into regular cages under 12/12 h light/dark cycle. The experimental procedures involving live animals were performed in strict accordance with institutional guidelines in compliance with national (D.L. N.26, 04/03/2014) and international law and policies (new directive 2010/63/EU). The study was approved by the Italian Ministry of Health (protocol #980/2020-PR, title “Evaluation of the effectiveness of SMN-dependent and SMN-independent therapies to counteract Spinal Muscular Atrophy”). Additionally, the ad hoc Ethical Committee of the University of Turin approved this study. Animal care and all experimental procedures were conducted following the ARRIVE guidelines. The research complies with the commonly accepted “3Rs”, minimizing the number of animals used and avoiding their suffering. A total number of 43 SMA and 15 WT mice were used; no criteria for including/excluding animals during the experiment were used.

The mice were genotyped by PCR using DNA isolated from tail snips collected at P0-P1, before starting the ASC-EVs/PBS treatment. Isolation was performed with proteinase k (50 µg) in lysis buffer (10mM Tris HCl, 50mM KCl, 0.01% gelatin, 0.45% IGEPAL, 0.4% Tween-20) at 60 °C for 30 min under gently shaking. The presence of the transgene was determined by PCR analysis using primers that amplify a portion of the *smn* gene, yielding a 420 bp product for the wild-type allele and a 150 bp product for the transgenic one. They were: *smn* fwd 5′- TTTTCTCCCTCTTCAGAGTGAT-3′, *smn* wt rev 5′- CTGTTTCAAGGGAGTTGTGGC-3′ and *smn* tg rev 5′- GGTAACGCCAGGGTTTTCC-3′ as suggested by suppliers (Jackson Laboratories). Genotyped mice were distinguished with a marker sign on their skin.

### ASC-EVs administration

To evaluate the therapeutic effects of ASC-EVs SMA mice were divided randomly into ASC-EVs-treated group (SMA-EVs) and PBS-treated group (SMA-PBS) (*n* = 16 SMA-EVs, 27 SMA-PBS). An additional group of wildtype (WT) pups not treated with ASC-EVs/PBS was considered as a healthy control group (*n* = 15 WT). ICV injections were performed at postnatal day 3 (P3) and P6: treated animals received 0.5 µg of ASC-EVs (injected volume 2 µl), while SMA control group was injected with 2 µl of PBS. Both ASC-EVs and PBS were at room temperature when administered.

Briefly, pups were anesthetized by hypothermia (total duration 3–5 min) and their heads were immobilized on a custom neonatal stereotaxic apparatus. ASC-EVs or PBS were injected with a Hamilton microsyringe alone, at stereotaxic coordinates of 0 mm from bregma, + 0.8 mm (at P3)/-0,9 mm (contralateral ventricle at P6) to sagittal sinus, and 1.5 mm deep, as reported in [15]. Pups were then placed on a heat pad, quickly revitalized and their vital functions were monitored. Then, they were returned to their mother after a short period of re-acclimatisation in their cage’s litter, assuring that their mother was not neglecting them. Pups wellbeing and behavioural conditions were inspected in the following hours and days.

### Behavioural and motor test

The symptom progression was monitored in SMA and WT mice by checking the body weight and by performing behavioural tests specifically designed for neonatal rodents [50]. SMA-PBS, SMA-EVs treated and WT animals (*n* = 16 SMA-EVs; 23 SMA-PBS; 7 WT) were tested at different time points: P2, P4, P6, P8 and P10. In particular, three behavioural tests were performed on pups: (I) Tail suspension test: to evaluate hindlimb posture and strength, pups were suspended by the tail for 15 s and a score was assigned to their hindlimb posture as follow: normal hindlimb spread open (score 4); not completely hindlimb spread open (score 3); hindlimb often close (score 2); hindlimb always close together (score 1); hindlimb always close together with clasping (score 0). (II) Righting reflex: pups were placed on their backs on a flat surface and their failure or success in repositioning themselves on dorsal side up was evaluated within 30 s. (III) Negative Geotaxis: for the evaluation of motor coordination and vestibular sensitivity, pups from P4 were placed on an inclined surface (approximately 35° inclination) with the head facing down. The ability of the pups to turn around and climb upwards was evaluated within 60 s and recorded.

### Tissue preparation

The humane endpoints were established and monitored in accordance with the Ethical Committee of the University of Turin. No mice exhibited any signs of the established endpoints in this study. For the histological analysis of brain (*n* = 2 WT), spinal cord (*n* = 5 SMA-EVs; 6 SMA-PBS; 3 WT), gastrocnemius and quadriceps muscles (*n* = 5 SMA-EVs; 7 SMA-PBS; 6 WT), at P10 animals were deeply anesthetized by gaseous anaesthesia (isoflurane 5%). After confirming that the animals had reached a state of deep anesthesia, pups were transcardically perfused with phosphate buffer 0.1 M (PB, pH 7.4), followed by paraformaldehyde (PFA) 4%, as described in [51] and reported in [52]. The tissues were dissected out and post-fixed in PFA 4% overnight at 4 °C. They were then soaked in 30% sucrose solution overnight, embedded and frozen in cryostat medium (Killik, Bio-Optica, Milan, Italy).

First of all, in order to exclude brain damages due to ICV injections and assess the proper targeting of the cerebral ventricles, the PFA 4%-fixed WT brains (P10) were dissected and cut in 40 μm-thick sections, subsequently mounted on 4% gelatin-coated glasses. The lumbar spinal cord (L1-L5) was cut in 40 μm thick, serial, free-floating sections that were stored in an antifreeze solution (30% ethylene glycol, 30% glycerol, 10% PB; 189 mM NaH2PO4; 192.5 mM NaOH; pH 7.4) and stored at -20 °C until being used. The gastrocnemius and quadriceps muscles were longitudinally cut in 20 μm sections with cryostat apparatus and directly collected on 4% gelatin-coated glasses.

For hematoxylin-eosin (H/E) staining of gastrocnemius and quadriceps muscles, another cohort of pups (*n* = 6 SMA-EVs; 5 SMA-PBS; 6 WT) were sacrificed by cervical dislocation at P10. Fresh muscles were rapidly collected, embedded in cryostat medium and cut at the cryostat in 20 μm thick transverse slices and collected directly onto 4% gelatin-coated glasses.

### Histochemistry

For Nissl staining, brain and spinal cord sections were mounted on 4% gelatin-coated slides and air-dried overnight. Sections were then hydrated in distilled water, immersed in 0.1% Cresyl violet acetate (Sigma Aldrich) for 5 min and gradually placed into increasing concentrations of ethanol, cleared with xylene, and cover-slipped with Eukitt (Bio-Optica). For H/E staining, sections of gastrocnemius and quadriceps muscle were stained firstly with hematoxylin, then with eosin (Bio-Optica), dehydrated in increasing concentrations of ethanol, cleared in xylene and cover-slipped with Eukitt (Bio-Optica).

### Immunohistochemistry

For immunofluorescence staining, the lumbar spinal cord sections were rinsed with PBS and incubated in blocking solution with 10% normal donkey serum (NDS) and 0.3% Triton X-100 in PBS for 30 min at RT. The sections were incubated overnight at 4 °C with the following primary antibodies diluted in 0.3% Triton X-100 and appropriate 2% normal sera in PBS: anti-Cleaved Caspase-3 (1:200, Asp175, rabbit, Cell Signaling Technology, Danvers, MA, USA), anti-SMI32 (1:1000, mouse, BioLegend, San Diego, CA, USA), anti-glial fibrillary acidic protein (anti-GFAP, 1:500, rabbit, DAKO Cytomation, Agilent, Santa Clara, CA, USA), anti-ionized calcium binding adaptor molecule 1 (anti-IBA1, 1:1000, rabbit, Wako Chemicals, Neuss, Germany).

After rinsing, primary antibodies were detected with appropriate fluorochrome-conjugated secondary antibodies (all from Jackson ImmunoResearch Laboratories, West Grove, PA, USA): Alexafluor anti-rabbit 647 (1:600), Cyanin-3 AffiniPure anti-rabbit (1:200) and Cyanin-2 AffiniPure anti-mouse (1:200) diluted in PBS for 1 h at RT. The sections were mounted on 4% gelatin-coated slices and coverslipped with the mounting medium Mowiol.

For the labelling of neuromuscular junctions (NMJs) and the following analysis of their innervation, the gastrocnemius and quadriceps muscles slices longitudinally cut were incubated for 30 min at room temperature with the Alexafluor-555-conjugated bungarotoxin (αBGTX, 1:500, Invitrogen, Milan, Italy), and overnight at 4 °C with primary antibody anti-neurofilament (anti-NF, 1:200, mouse, 2H3 clone, Hybridoma Bank, Iowa, IA, USA), diluted in 0.3% Triton X-100 in PBS and detected with Cyanin-2 AffiniPure anti-mouse (1:200; Jackson ImmunoResearch Laboratories, West Grove, PA, USA) secondary antibody. The slices were coverslipped with the mounting medium Mowiol.

Immunoreacted spinal cord and skeletal muscles were then analysed with a Leica TCS SP5 confocal laser scanning microscope (Leica Microsystems) or with Nikon Eclipse E90i epifluorescence microscope.

### Quantitative analysis

For the MNs counting (*n* = 5 SMA-EVs; 6 SMA-PBS; 3 WT), Nissl stained-MNs in the ventral horns of the lumbar spinal cord were stereologically counted at 40X (one every fifth 40 μm-thick section was reconstructed) using a stereological technique with a computer-assisted microscope and the StereoInvestigator software (MicroBrightField Inc., Williston, VT, USA). MNs were counted when characterized by a diameter of 10 μm and located in the ventral somatic columns. The cell density was reported as MNs number/mm^3^.

The Cleaved Caspase-3-positive L1-L5 MNs (*n* = 4 SMA-EVs; 6 SMA-PBS; 3 WT) were quantified by counting the percentage of Casp3+/SMI-32 + double labelled cells on the total SMI32 + MNs cell population, using confocal images of the ventral horns of the lumbar spinal cord. Three spinal cord slices were evaluated for each animal.

For the analysis of astrogliosis in the ventral horn of the lumbar spinal cord, confocal images of GFAP-positive cells (*n* = 4 SMA-EVs; 6 SMA-PBS; 3 WT) and IBA-1-positive cells (*n* = 5 SMA-EVs; 6 SMA-PBS; 3 WT) were converted in black and white images, then the density of immunopositive profiles on the total area was quantified using Image J and expressed as a percentage.

Furthermore, the IBA-1-positive cells were qualitatively classified by their shape in ramified, bushy or amoeboid based on the morphology phenotypes described in [53, 54] and the results were expressed as a percentage on the total number of IBA-1-positive cells.

To morphologically evaluate the gastrocnemius and quadriceps muscles, H/E stained muscle sections were visualized by optical microscopy (Olympus BX63; Olympus Life Science Solutions, Center Valley, PA (*n* = 6 SMA-EVs; 5 SMA-PBS; 6 WT): to measure the mean fibers area and Feret’s max diameter, the sections were drawn and analysed by Image J software. A total of 100 fibers per quadriceps and gastrocnemius were drawn and analysed for each animal.

Finally, for the analysis of NMJ innervation in gastrocnemius and quadriceps muscles for each animal (*n* = 5 SMA-EVs; 7 SMA-PBS; 6 WT) at least 100 NMJs/muscle were analysed with Nikon Eclipse E90i epifluorescence microscope; each NMJ was then classified in innervated, multi-innervated or denervated by looking at the number of NFs contacting the endplate and the result was expressed as a percentage on the total number of NMJs.

### Statistical analyses

All the data were expressed as mean ± standard error of the mean (SEM). Data were analysed using Two-way ANOVA (for the behavioural results) and One-way ANOVA or Student t-test (for the immunofluorescence and histological analysis): p-values < 0.05 were considered significant.

## Results

### Isolation and characterisation of ASC-EVs

EVs were isolated from ASCs supernatant with an EVs isolation kit. The protein concentration was quantified and the yield of proteins for each isolation was about 200 µg/mL.

**Fig. 1:**
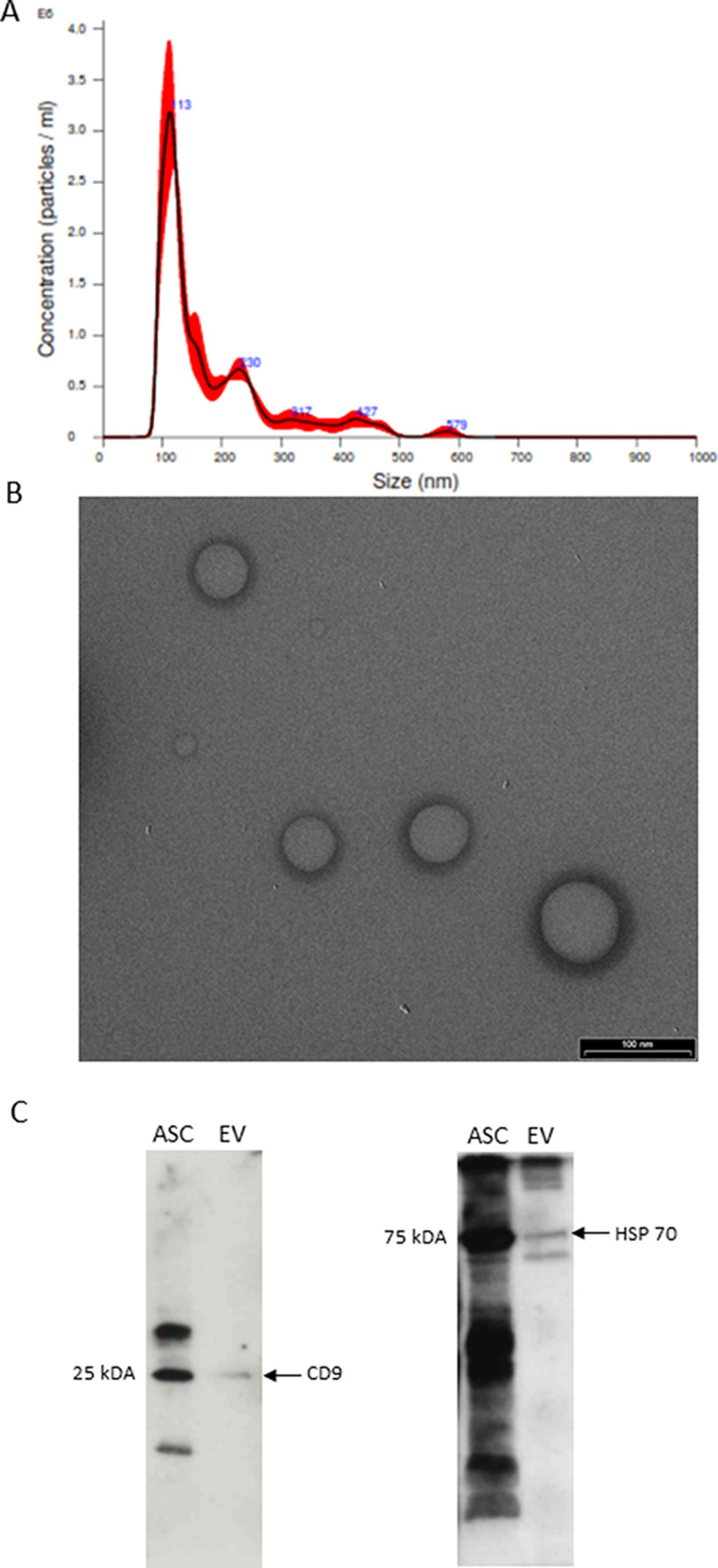
Characterization of ASC-EVs. (**A**) ASC-EVs size and concentration were measured and analysed by NTA. (**B**) Representative transmission electron microscopy images of ASC-EVs showed particles with characteristic morphology and size (scale bar 100 nm). (**C**) Western blot analysis of specific EVs protein markers: bands at 25, and 75 kDa were present after incubation with CD9 and HSP70 antibodies, respectively. ASCs lysates were used as positive control

ASC-EVs were analysed and quantified by NTA: the concentration of EVs was 2.38 × 10^8^ particles/mL, with a particle diameter mode of 113.6 nm (Fig. 1a; Table 1). Ultrastructure analysis of the EVs by TEM revealed round vesicles with lipid bilayers with a diameter of 50 to 150 nm (Fig. 1b). Measurements are in line with literature [32]. By western blot we validated the presence of typical markers of EVs [55] identified through CD9 and HSP-70 antibodies, that displayed specific signals at 25 and at 75 kDa respectively (Fig. 1c). These results confirm that size, morphology, and the presence of specific protein markers are consistent with EVs.

Moreover, in a previous study regarding the proteomic analysis of ASC-EVs [56] the FL-SMN protein was not present in the extracellular vesicles content, supporting the idea that this could act as a SMN-independent treatment.

**Table 1:** NTA data output which reports mean and mode of ASC-EVs size and particles concentration with relative SD.

Size of ASC-EVs			
Mean (nm)	Mode (nm)	SD (nm)	
181.7 ± 5.8	113.6 ± 6.7	101.2 ± 8.4	
**Concentration of ASC-EVs**			
Concentration (particles/ml)		Concentration (particles/frame)	Concentration (centres/frame)
2.38 × 10^8^ ± 2.31 × 10^7^		36.0 ± 2.4	52.0 ± 3.3

### ASC-EVs treatment improves the disease progression in SMA mice

At P3 and P6, SMA pups received two ICV administrations of ASC-EVs or PBS (2 µl). As expected, this procedure did not cause any damage to the cerebral cortex and ventricles (Suppl. Figure 1). Moreover, the injections were well tolerated by SMA mice.

**Fig. 2:**
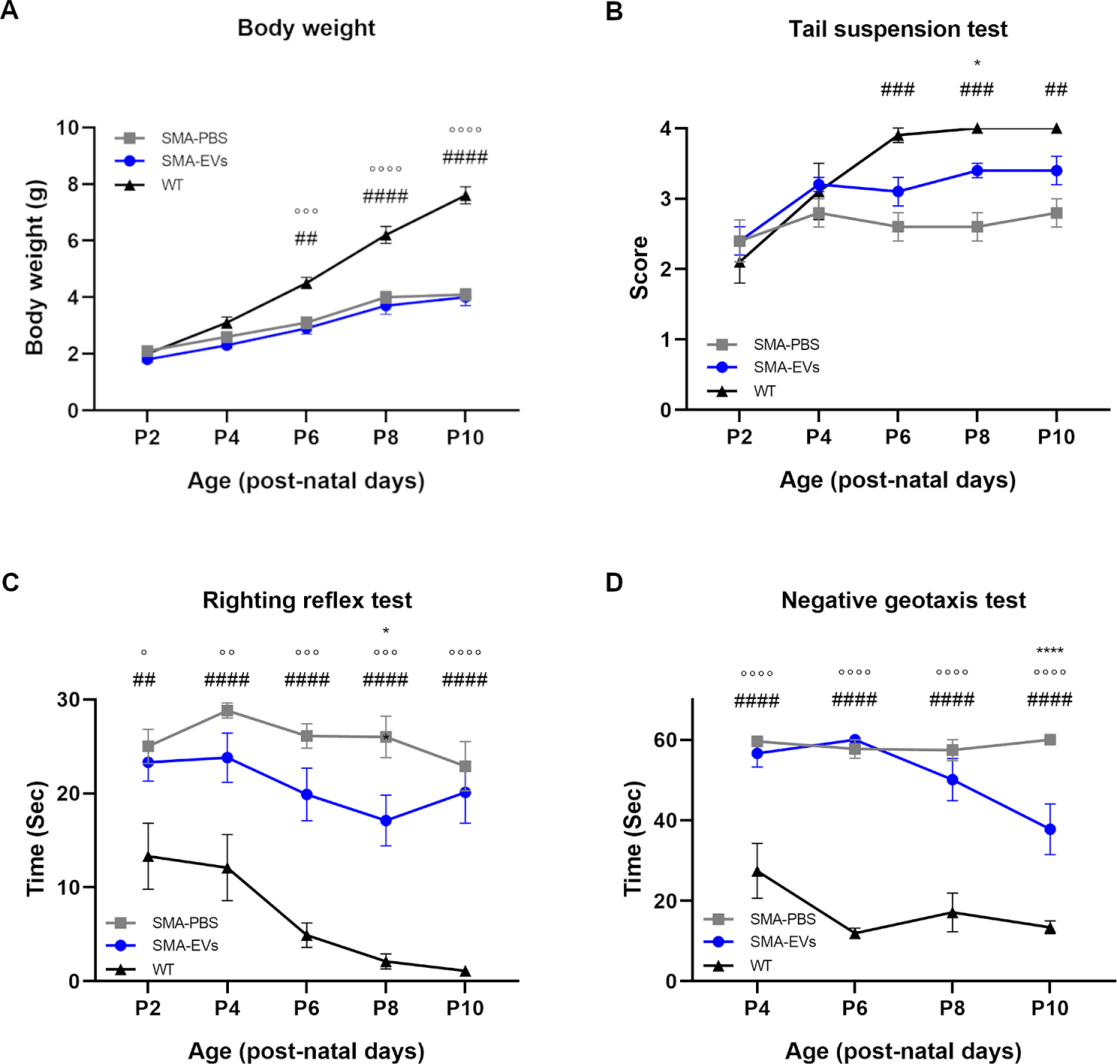
ASC-EVs treatment improves the motor performance of SMA mice. From P2/P4 to P10 the body weight measurements (**A**), tail suspension test (**B**), righting reflex test (**C**) and negative geotaxis test (**D**), were used to evaluate the motor performance of SMA mice treated with PBS (SMA-PBS, grey line) or ASC-EVs (SMA-EVs, blue line) and WT mice (black line). Overall, the results suggest an improvement in behavioural and motor performances of SMA-EVs mice compared to the SMA-PBS group

During the entire treatment period up to the sacrifice, SMA and WT mice body weight (Fig. 2a) was monitored, showing no differences between SMA-PBS and SMA-EVs group, while differences were observed between SMA-PBS and WT animals (P6 ##*p* = 0.0014; P8, P10 ####*p* < 0.0001) and between SMA-EVs and WT animals (P6 °°°*p* = 0.0004; P8, P10 °°°°*p* < 0.0001). However, the body weight increase of SMA-PBS group reached the maximum value at P8, while that of SMA-EVs mice was still increasing at P10 and overall, the statistical analysis showed a significant treatment (F_(2, 175)_ = 71.65, ****p < 0.0001), time (F_(4, 175)_ = 110.1, ****p < 0.0001) and interaction (F_(8, 175)_ = 10.29, ****p < 0.0001) effect.

To investigate the effect of ASC-EVs treatment, pups underwent a battery of behavioural tests from P2/P4 to P10: tail suspension, righting reflex and negative geotaxis tests.

In the tail suspension test (Fig. 2b) the hindlimb posture and strength were evaluated: after the injections at P3 and P6 SMA-EVs mice obtained higher scores compared to SMA-PBS mice, showing a statistical difference at P8 (*p = 0.0251). Compared to the WT group, SMA-PBS showed statistical differences at P6, P8 ###*p* = 0.0004) and P10 ##*p* = 0.0028), while no differences were observed for SMA-EVs group. The statistical analysis showed a significant treatment (F_(2, 175)_ = 14.33, ****p < 0.0001), time (F_(4, 175)_ = 10.62, ****p < 0.0001) and interaction (F_(8, 15)_ = 2.34, * *p* = 0.0208) effect.

The righting reflex test (Fig. 2c) was also performed and results revealed that SMA-EVs group could complete the task in a shorter time compared to SMA-PBS group, already from P4; in particular, at P8 (after the second ASC-EVs injection, P6) the performance of the two SMA groups appeared significantly different (P8 *p = 0.0167). Statistical differences where observed between SMA-PBS and WT animals (P2 ##*p* = 0.0057; P4, P6, P8, P10 ####*p* < 0.0001) and also between SMA-EVs and WT mice (P2 °*p* = 0.0218; P4 °°*p* = 0.0057; P6 °°°*p* = 0.0003; P8 °°°*p* = 0.0004; P10 °°°°*p* < 0.0001) Moreover, the statistical analysis showed a significant treatment (F_(2, 175)_ = 67.24, ****p < 0,0001) and time (F_(4, 175)_ = 4.83, **p = 0,0010) effect while the interaction between treatment and time was not significant (F_(8, 175)_ = 1.12, *p* > 0,05) .

Regarding the negative geotaxis test (Fig. 2d), which measures the strength and motor coordination of pups, the control SMA group showed difficulties in completing the test during the whole observation period, while the treated one gradually ameliorated their motor performances, that were statistically different at P10 compared to the SMA-PBS group (P10 ****p < 0.0001). Differences between SMA-PBS/SMA-EVs and WT group were observed along all the observation period (P2, P4, P6, P8, P10 ****p < 0.0001). Overall, a significant treatment (F_(2, 137)_ = 113.2, ****p < 0.0001), time (F_(3, 137)_ = 4.15, **p = 0.0075) and interaction between treatment and time (F_(6, 137)_ = 3.52, **p = 0.0029) effects were observed.

All these data suggest an overall improvement in motor performances of SMA-EVs mice compared to the SMA-PBS group, in particular after the second ASC-EVs injection, even if a complete rescue to the healthy condition was not reached.

Data are shown as mean ± SEM and were analysed by Two-way ANOVA mixed-effects model with Geisser-Greenhouse correction followed by Sidak’s multiple comparison post hoc test. Statistical difference between the groups are indicated (*p < 0.05; **p < 0,005; ***p < 0,0005; ****p < 0.0001).

Legend: * = SMA-EVs vs. SMA-PBS; ° = SMA-EVs vs. WT; # = SMA-PBS vs. WT.

### ASC-EVs administration extends the survival of lumbar MNs in SMA mice

**Fig. 3:**
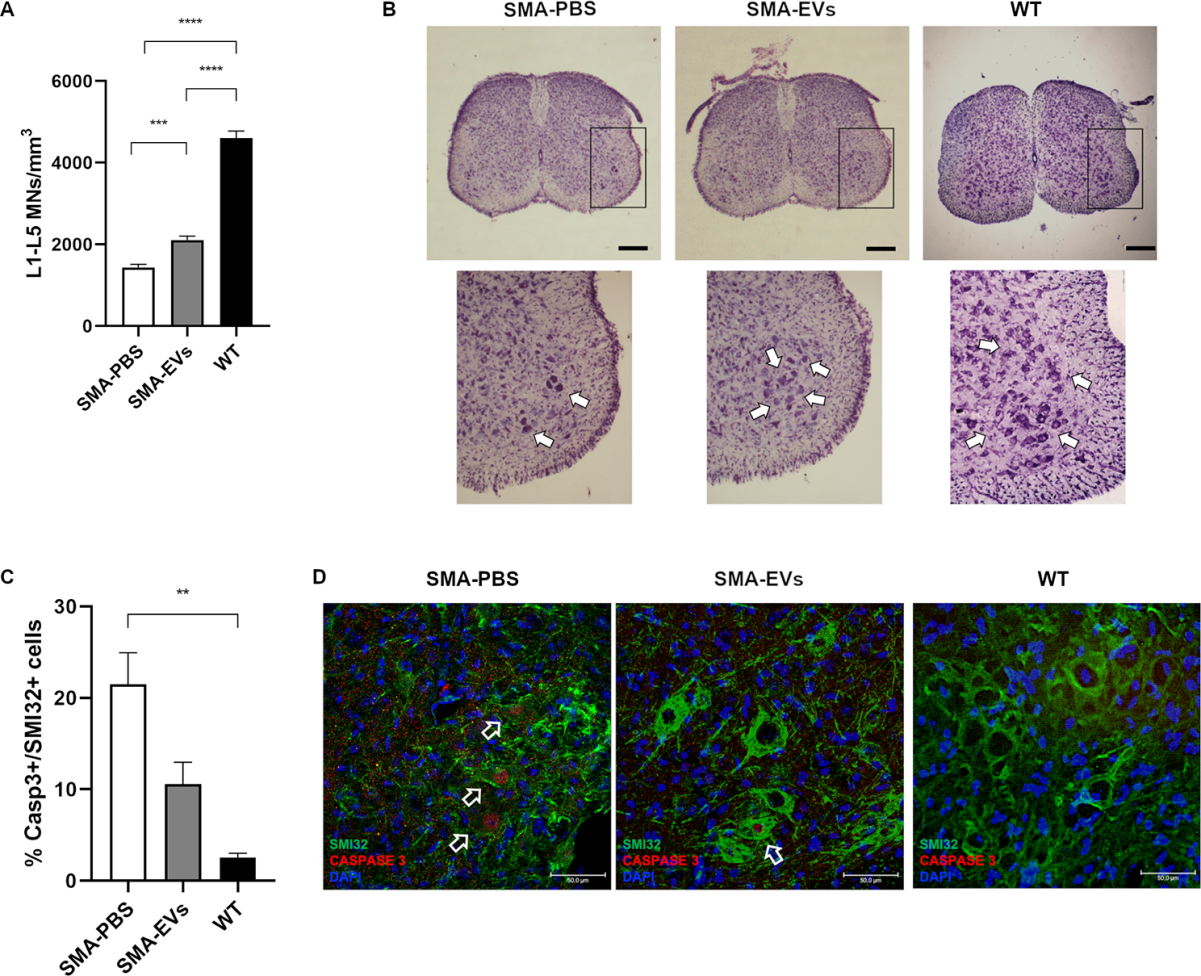
Effect of ASC-EVs administration on lumbar MNs degeneration in SMA mice. (**A**) The graph shows the quantification of MNs density (MNs number/volume) in the ventral horns of L1-L5 spinal cord for SMA-PBS (white), SMA-EVs (grey) and WT (black) group at P10. The treatment with ASC-EVs significantly protect MNs from neurodegeneration compared to control mice (One-way ANOVA ***p < 0.0010; ****p < 0.0001). (**B**) Representative Nissl stained sections of lumbar spinal cord of SMA-PBS, SMA-EVs and WT mice. The arrows show stained MNs. Scale bar 500 μm. (**C**) The graph shows the quantification of the percentage of MNs (SMI32 + cells) expressing Casp3 in SMA-PBS (white), SMA-EVs (grey) and WT (black) groups: the treatment with ASC-EVs decreased the activation of apoptotic marker Casp3 compared to control mice (One-way ANOVA **p = 0.0050). (**D**) Representative confocal images showing Casp3+ (red) and SMI32+ (green) cells in the ventral horns of PBS- and ASC-EVs treated SMA and WT mice. Cell nuclei are labelled by DAPI staining (blue). The arrows show Casp3+/SMI32 + cells. Scale bar 50 μm

At P10 the animals were sacrificed, lumbar spinal cords were dissected and analysed to evaluate the neuroprotective effect of ASC-EVs treatment on SMA mice. In particular, a stereological count of MNs was performed in the ventral horns of the lumbar tract (L1-L5) in all the experimental groups. The results highlighted a significantly higher MN density in the lumbar spinal cord of SMA-EVs animals (2106.87 ± 96.47 MNs/mm^3^) compared to the SMA-PBS counterpart (1438.80 ± 73.09 MNs/mm^3^; ***p = 0.0010.). WT animals showed an expected significant higher MN density (4604.97 ± 173.24 MNs/mm^3^) compared to SMA-PBS and SMA-EVs groups (****p < 0.0001. Figure 3a,b).

To further investigate the neuroprotective action of the ASC-EVs treatment in the SMA-EVs group, we then consider in the ventral horn of the lumbar spinal cord sections (L1-L5) the expression of the apoptotic marker Cleaved Caspase-3 (Casp3). The percentage of Casp3+/SMI32 + co-labeled MNs quantified by immunohistochemical analysis was reduced in SMA mice treated with ASC-EVs (10.59 ± 2.36%) compared to the SMA-PBS group (21.48 ± 3.47%). WT animals showed a statistically lower expression of Casp3 (2.51 ± 0.50%) compared to SMA-PBS group (**p = 0.0056), while importantly no statistical differences were observed compared to SMA-EVs animals (Fig. 3c,d). However, considering the effect of ASC-EVs treatment compared to control alone, a significant difference between SMA-PBS and SMA-EVs animals is present (Unpaired t test *p < 0.05).

All together, these data suggest that the ASC-EVs administration is able to protect the lumbar MNs from degeneration.

### ASC-EVs treatment modulates the neuroinflammation in SMA mice

Since, besides MNs degeneration, neuroinflammation is reported in SMA as well [57], we evaluated by immunohistochemical analysis the activation rate of astrocytes and microglia in the ventral horns of the lumbar spinal cord sections of P10 SMA and WT animals.

**Fig. 4:**
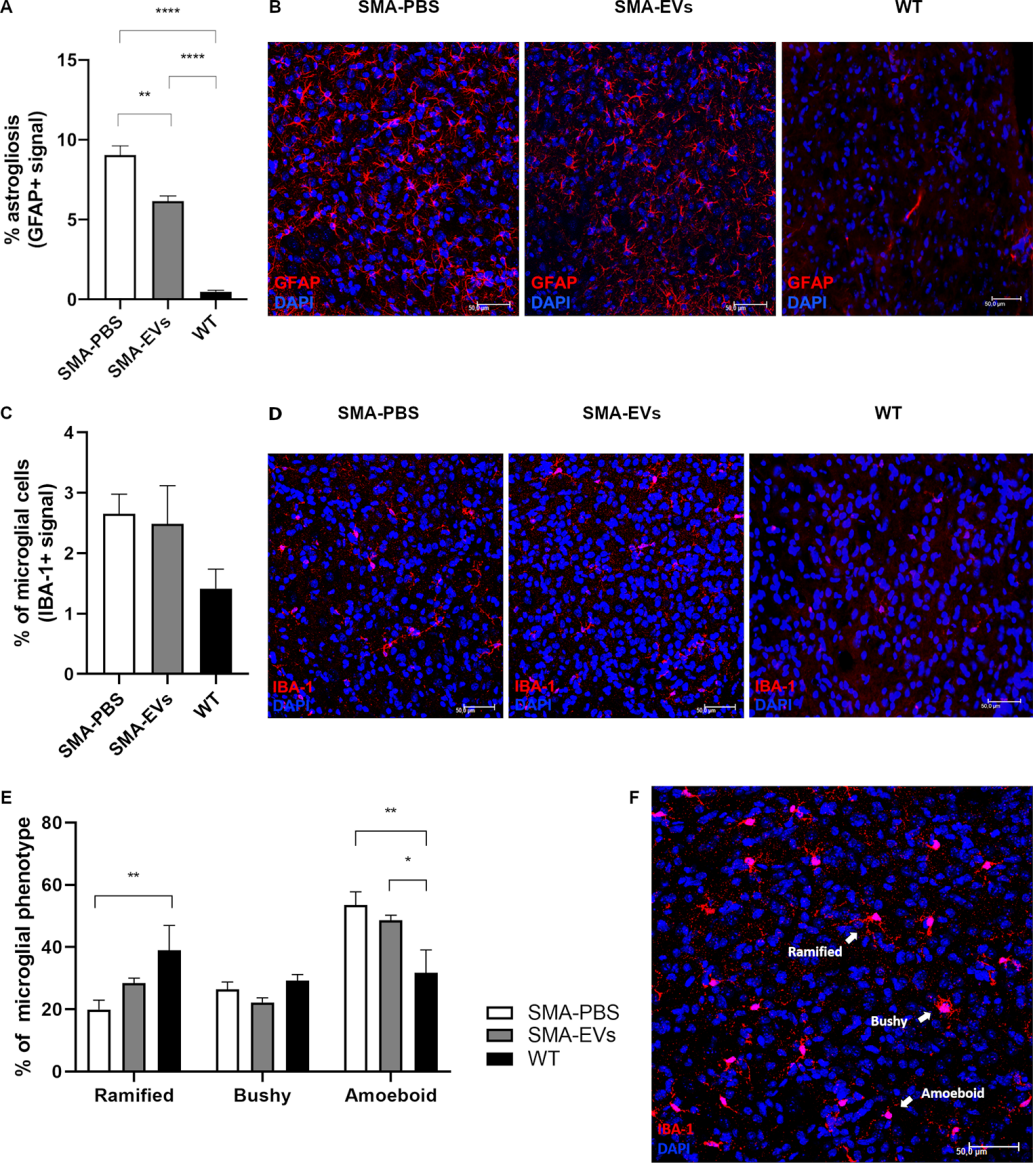
ASC-EVs treatment modulates the neuroinflammation in SMA mice. (**A**) The graph shows the quantification of the percentage of astrogliosis (GFAP + signal) in the ventral horns of L1-L5 spinal cord for SMA-PBS (white), SMA-EVs (grey) and WT (black) mice at P10. The ASC-EVs administration significantly decreased the percentage of GFAP-immunopositive profiles in SMA-EVs mice compared to SMA-PBS ones (One-way ANOVA **p = 0.0040; ****p < 0,0001). (**B**) Representative confocal images showing GFAP+ (red) cells in the ventral horns of PBS- (SMA-PBS) and ASC-EVs treated (SMA-EVs) SMA mice and WT mice. Cell nuclei are labelled by DAPI staining (blue). Scale bar 50 μm. (**C**) The graph shows the quantification of the percentage of microglial cells (IBA-1 + signal) in the ventral horns of L1-L5 spinal cord for SMA-PBS (white), SMA-EVs (grey) and WT (black) mice at P10. No differences in the percentage of IBA-1 immunopositive profile were observed between SMA-PBS, SMA-EVs and WT mice (One-way ANOVA *p* > 0.05). (**D**) Representative confocal images showing IBA-1+ (red) cells in the ventral horns of PBS- (SMA-PBS) and ASC-EVs treated (SMA-EVs) SMA mice and WT mice. Cell nuclei are labelled by DAPI staining (blue). Scale bar 50 μm. (**E**) The graph displays the microglial cell classification in ramified, bushy or amoeboid, based on their shape; the results are expressed as a percentage on the total number of IBA-1-positive cells for SMA-PBS (white), SMA-EVs (grey) and WT (black) group (Two-way ANOVA *p < 0.05; **p < 0.0050). (**F**) Representative confocal images showing IBA-1+ (red) microglial cells “ramified”, “bushy” and “amoeboid” in the ventral horns of spinal cord. Cell nuclei are labelled by DAPI staining (blue). Scale bar 50 μm

In particular, our results showed that ASC-EVs treatment could significantly reduce the GFAP expression (SMA-EVs 6.16 ± 0.315%) compared to the SMA-PBS controls (9.06 ± 0.566%; **p = 0.0040). WT animals reported a lower GFAP expression (0.48 ± 0.089%), statistically different compared to SMA-PBS and SMA-EVs groups (****p < 0.0001; Fig. 4a,b).

The same analysis was carried out also to evaluate the activation of the microglia in the lumbar spinal cord of SMA and WT mice; however, no differences in the IBA-1 + signal were observed between SMA-PBS (2.66 ± 0.324%), SMA-EVs (2.49 ± 0.631%) and WT (1.41 ± 0.325%) mice (*p* > 0.05. Figure 4c,d). We also evaluated the morphology of microglial cells, in order to preliminary correlate their cellular phenotype with their function. Microglial cells were classified based on their shape in ramified, bushy or amoeboid (Fig. 4e) and the results were expressed as a percentage on the total number of IBA-1-positive cells. The outcomes did not show any statistical differences between SMA-PBS and SMA-EVs, but a higher percentage of the “ramified” phenotype (typical of a steady-state microglial cells) was observed for the SMA-EVs group (28.41 ± 1.64%) compared to the SMA-PBS one (19.94 ± 3.03%). Also considering the “bushy” and “amoeboid” phenotypes, there were no statistical differences between SMA-PBS (bushy: 26.46 ± 2.32%; amoeboid: 53.59 ± 4.18%) and SMA-EVs pups (bushy: 22.22 ± 1.46%; amoeboid: 48.63 ± 1.59%), but these percentages were higher for the SMA-PBS group. However, taking into account the effect of ASC-EVs treatment compared to the control group alone, a significant difference between SMA-PBS and SMA-EVs animals is present for the “ramified” phenotype (Unpaired t test *p < 0.05). Comparing to WT group (ramified: 39.00 ± 8.00%; bushy: 29.22 ± 1.92%; amoeboid: 31.79 ± 7.32%) statistical differences were observed for SMA-PBS group (ramified **p = 0.0089; amoeboid **p = 0.0021) and for SMA-EVs group (amoeboid *p = 0.0357; Fig. 4e,f).

These data suggest that ASC-EVs treatment is able to modulate the neuroinflammation in SMA mice, possibly contributing to delay the MN death.

### The effects of ASC-EVs administration on skeletal muscles atrophy and NMJ maturation

Given the encouraging results observed with the motor tests and in the lumbar spinal cord in SMA mice after ASC-EVs administration, we hypothesized that the central effects of our treatment could in turn positively affect also the muscular trophism and innervation at peripheral level.

**Fig. 5:**
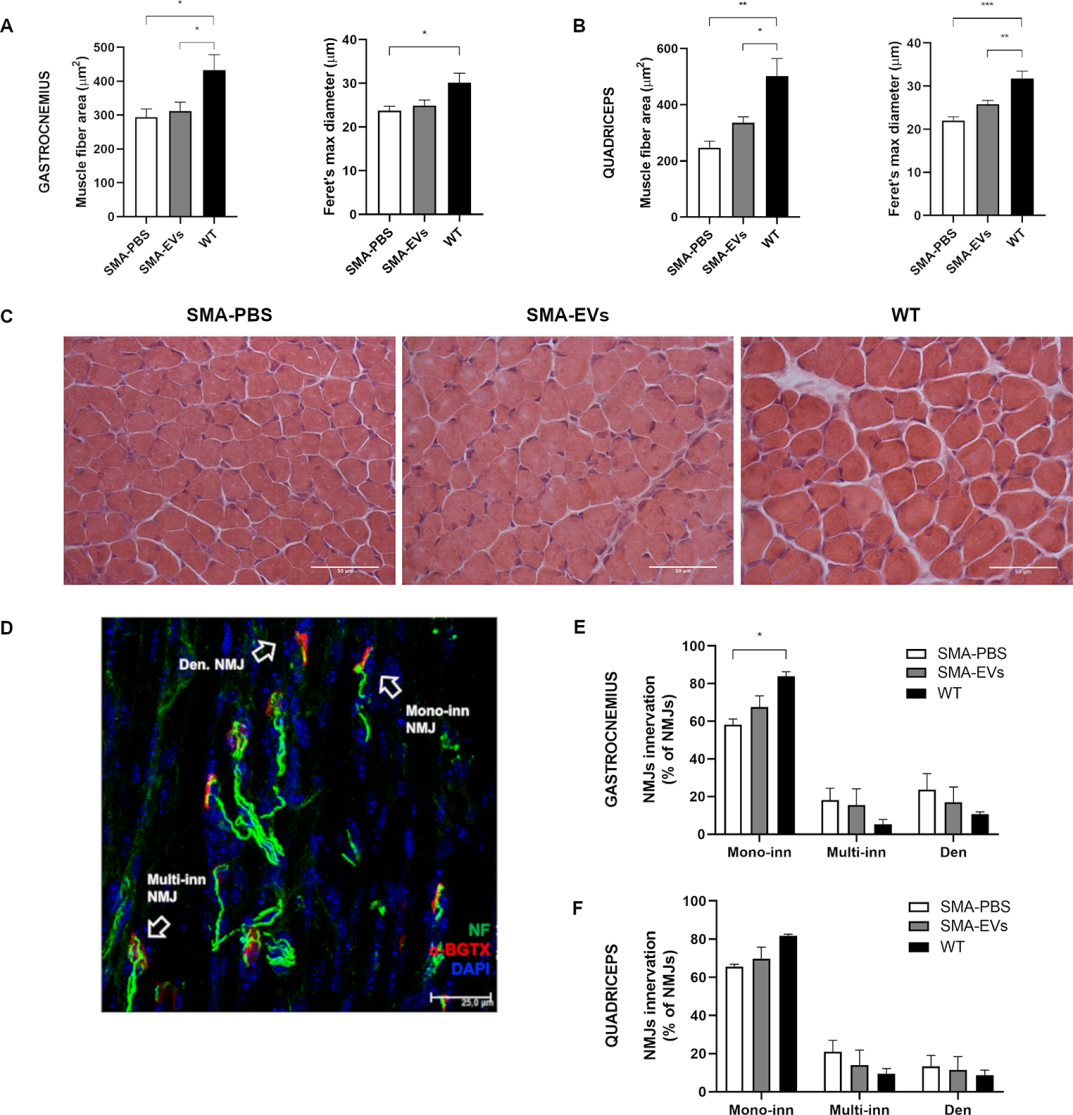
Effect of ASC-EVs treatment on skeletal muscles of SMA mice. The graphs show the quantitative analysis of the mean fiber area and Feret’s max diameter of gastrocnemius muscle (**A**) and quadriceps muscle (**B**) for SMA mice treated with PBS (SMA-PBS, white) or with ASC-EVs (SMA-EVs, grey) and for WT mice (black). ASC-EVs treatment can partially rescue the atrophy of skeletal muscle fibers in SMA mice. (One-way ANOVA *p < 0.05; **p < 0,005; ***p < 0,0005). (**C**) Hematoxylin/eosin (H/E) stained representative images showing SMA-PBS, SMA-EVs and WT quadriceps fibers. Scale bar 50 μm. (**D**) Representative confocal image showing NMJs (αBGTX, red; NF-H, green, DAPI blue) in quadriceps muscle of SMA-PBS. The arrows show three different NMJs phenotypes: mono-innervated (a single NF is contacting the endplate), multi-innervated (several NFs are contacting the endplate) and denervated (no co-localization between NF-H and αBGTX). Scale bar 25 μm. The graphs show the quantification of the percentage of mono-innervated, multi-innervated and denervated NMJs in gastrocnemius muscle (**E**) and quadriceps muscle (**F**) in SMA mice treated with PBS (SMA-PBS, white) or with ASC-EVs (SMA-EVs, grey) and in WT mice (black). Statistical difference between the groups are indicated (Two-way ANOVA *p < 0.05)

Therefore, we firstly analysed the morphology of gastrocnemius and quadriceps fibers at P10 in terms of mean fibers area and Feret’s max diameter. Regarding the gastrocnemius, the results did not show any differences both in the mean fibers area and in the Feret’s max diameter between SMA-PBS (fibers area: 294.24 ± 23.92 µm^2^; Feret’s max diameter: 23.75 ± 1.00 μm) and SMA-EVs groups (fibers area: 311.81 ± 26.34 µm^2^; Feret’s max diameter: 24.93 ± 1.27 μm), while there were statistical differences between WT (fibers area: 432.57 ± 45.55 µm^2^; Feret’s max diameter: 30.17 ± 2.15 μm) and SMA-PBS group (Mean fibers area *p = 0.0309; Feret’s max diameter *p = 0.0315) and between WT and SMA-EVs group (Mean fibers area *p = 0.0494. Figure 5a).

When considering the quadriceps muscle the morphological analysis did not show any differences both in the mean fibers area and in the Feret’s max diameter between SMA-PBS (fibers area: 247.23 ± 22.78 µm^2^; Feret’s max diameter: 22.01 ± 0.88 μm) and SMA-EVs groups (fibers area: 336.03 ± 21.06 µm^2^; Feret’s max diameter: 25.85 ± 0.87 μm). However taking into account the effect of ASC-EVs treatment compared to the control group alone, a significant difference between SMA-PBS and SMA-EVs animals is present both in the mean fibers area (Unpaired t test *p = 0.0188) and in the Feret’s max diameter (Unpaired t test *p = 0.0127). The analysis also highlighted differences between WT (fibers area: 502.93 ± 61.53 µm^2^; Feret’s max diameter: 31.76 ± 1.72 μm) and SMA-PBS group (Mean fibers area **p = 0.0013; Feret’s max diameter ***p = 0.0002) and between WT and SMA-EVs group (Mean fibers area *p = 0.0195; Feret’s max diameter **p = 0.0089. Figure 5b,c).

Secondly, we assessed the analysis on NMJs innervation and maturation by immunohistochemical reaction and we classified them as mono-innervated, multi-innervated or denervated by looking at the number of NFs contacting the endplate (Fig. 5d). At P10 in the gastrocnemius muscle we observed an increase in the percentage of mono-innervated NMJs in SMA-EVs mice, compensated by a decrease in the percentage of multi-innervated and denervated NMJs (SMA-EVs mono-inn.: 67.54 ± 5.94%; multi-inn.: 15.44 ± 8.69%; den.: 17 ± 8.16%) compared to SMA-PBS controls (mono-inn.: 58.17 ± 3.04%; multi-inn.: 18.1 ± 6.32%; den.: 23.74 ± 8.43%), even if no statistical differences were revealed (Fig. 5e).

Similarly, in the quadriceps muscle we observed an increase in the percentage of mono-innervated NMJs in SMA-EVs mice (mono-inn.: 69.78 ± 6.00%; multi-inn.: 13.94 ± 7.90%; den.: 11.42 ± 7.04%) compared to SMA-PBS controls (mono-inn.: 65.6 ± 1.24%; multi-inn.: 20.99 ± 5.99%; den.: 13.4 ± 5.63%), with no statistical difference between the two groups (Fig. 5f). The results showed instead significant differences between WT (Gastrocnemius mono-inn.: 83.98 ± 2.30%; multi-inn.: 5.32 ± 2.49%; den.: 10.68 ± 1.22%. Quadriceps mono-inn.: 81.75 ± 0.91%; multi-inn.: 9.50 ± 2.65%; den.: 8.73 ± 2.62%) and SMA-PBS animals in the mono-innervated classification of NMJs in the gastrocnemius muscle (*p = 0.0112; Fig. 5e,f).

Overall, these results suggest that ICV ASC-EVs treatment can also partially counteract the muscular atrophy, in particular of early SMA affected muscles as quadriceps.

## Discussion

The pathogenesis of SMA is linked to the deficiency of the full-length SMN protein due to deletion or mutation on the *SMN1* gene. Several SMN-dependent therapeutic strategies have been exploited to restore the level of SMN by correcting the *SMN2* gene splicing as both Nusinersen (Spinraza, Biogen) and Risdiplam (Evrysdi, Roche) [58, 59] or by delivering a functional copy of *SMN1* gene into cells for its expression, as Onasemnogene abeparvovec (Zolgensma, Novartis) [60]. Besides the remarkable results obtained with these approved drugs, some issues and limitations remain, such as still unknown long-term effects, the possible treatment-related toxicity, and the high cost of these therapies. Furthermore, another relevant limitation concerning SMN-dependent therapies is that these strategies overlook other molecular and cellular pathways involved in SMA pathogenesis. A way to overcome this problem could be to implement SMN-dependent therapies with SMN-independent ones to exploit their synergistic effect [61].

In this regard, stem cells therapy has the advantage of counteracting pathogenic pathways by modulating several molecular and cellular mechanisms, given their ability to reach damaged sites where they differentiate and stimulate tissue repair and regeneration. However, stem cells therapeutic effects were later demonstrated to be due to their indirect and paracrine effect obtained through the release of EVs, rather than to their engraftment in damaged tissues. Indeed, EVs are able to recapitulate the major advantages of their cells source transferring their cargo (protein, lipids, mRNAs and miRNAs) to nearby cells and avoiding all the risks associated to cells therapy as well [23, 62].

With all these characteristics, EVs are a promising tools for several neurodegenerative diseases [63]: as reported by Wang and colleagues MSC-derived EVs improved the cognitive impairments and reduced the hippocampal β-amyloid aggregation and neuronal loss in a murine model of Alzheimer disease [33]. Intravenous MSC-EVs administration improved motor deficits, reduced brain atrophy and modulated brain neuroinflammation in a progressive model of multiple sclerosis [34] and ameliorated chronic experimental autoimmune encephalomyelitis pathogenesis [64]. In previous studies we demonstrated that ASC-EVs exerted a neuroprotective effect in an in vitro model of ALS [40] as well as in the SOD1(G93A) murine model: indeed, we showed that repeated administration of ASC-EVs improved motor performance, protected lumbar MNs, NMJs and skeletal muscles and decreased the glial cell activation in treated animals [41].

In this study, we investigated whether ASC-EVs could ameliorate the progression of another motor neuron disease such as SMA, different from ALS for its etiopathogenesis but with several pathological mechanisms in common. Therefore, we treated SMA pups with ICV injections at two different time points (at P3 and P6) and sacrificed the animals at P10 to evaluate the effect of the treatment.

From P2 to P10, mice underwent a battery of motor tests to follow the disease progression: although the treatment of SMA pups with ASC-EVs could generally hardly restore them to the healthy condition represented by WT animals, due also to the intrinsic severe phenotype of the model, we observed that the treatment improved the motor performance of treated animals compared to the SMA-PBS group; in particular, after the second injection, we could appreciate major effects in the righting reflex, tail suspension and negative geotaxis tests.

At the lumbar level of spinal cord, ASC-EVs treatment efficiently counteracted the MN degeneration compared to the vehicle-treated group, possibly explaining the observed improved motor performances of treated mice. From our previous proteomic study we know the ASC-EVs content, in which we did not detect the presence of the FL-SMN protein, thus supporting the idea that ASC-EVs act as an SMN-independent treatment in SMA mice. On the other side, ASC-EVs content comprises several proteins able to influence different cellular pathways, as for example the apoptotic process: in particular, the analysis pointed out the presence of proteins involved in the PI3K-Akt signalling pathway like the insulin-like growth factor (Igf1) [56]. Indeed through this signalling pathway, Igf1 protein, after binding with its receptor Igf1R, activates Akt, which prevents the apoptosis by inhibiting the pro-apoptotic protein Bad and stimulates cells proliferation [65, 66]. Furthermore, it has been already demonstrated that PI3K/Akt pathway is affected in SMA animals [67] and Akt phosphorylation was found to be reduced in SMA mice MNs primary cultures [68]. Therefore, we evaluated the expression of the apoptotic marker Cleaved Caspase-3, that is upregulated in in vitro [68] and in vivo [15] SMA models. What we observed was a reduction of Cleaved Caspase-3-positive lumbar MNs in SMA-EVs animals compared to control and importantly no significant differences compared to the WT group, confirming the neuroprotection efficacy of ASC-EVs treatment.

As reported in literature, neuroinflammation is observed in SMA and can negatively influence the MN survival through the release of pro-inflammatory molecules [57]. Since SMN-deficient astrocytes showed alterations and impairments [69] as well as increased expression of GFAP [70], we analysed the activation of astrocytes in the lumbar spinal cord: the outcomes showed a significant reduction of the percentage of astrogliosis in the animals treated with ASC-EVs. Also microglia seems involved in the pathogenesis of SMA [13]: Tarabal and colleagues confirmed the presence of activated microglia in the lumbar spinal cord in a rodent SMA model [71]. In our study we could not observed any differences in the total number of activated microglia between control, treated and WT group. However, we performed a qualitative classification based on the shape of IBA-1 positive cells into ramified, bushy or amoeboid. We observed a higher number for the ramified phenotype (typical of steady-state microglial cells [53, 54] for the treated group compared to the control one. We also observed a decrease of the bushy and amoeboid phenotype (respectively representing an intermediate activation state with morphological transformations/de-ramification in response to neuro inflamed environment and a fully activated and phagocytic microglia), although not significant.

In addition, at the peripheral level we evaluated skeletal muscles that are known to show atrophy and denervation, also because of neurodegeneration [52] and the crosstalk between MNs and muscles. From the comprehensive analysis between SMA and WT animals we could not appreciate a significant impact of ASC-EVs treatment at peripheral level. However, from a morphological point of view (muscle fibers area and Feret maximum diameter), quadriceps muscle of treated group showed a trend of muscle atrophy reduction compared to control group, although not statistically significant. This could be in line with other studies that demonstrated that quadriceps is early affected during the disease progression [72] as a consequence of selective vulnerability of the MNs innervating proximal muscles [73]. Therefore, it could be possible that the ASC-EVs neuroprotective effect on MNs through the delivery of molecules and proteins involved (for example in cell proliferation and angiogenesis [56]) could consequently positively influence in part also the muscular trophism. In SMA mouse models (and in human patients as well) NMJs developmental impairment is also reported including several hallmarks such as immaturity, denervation and NF aggregation [74, 75]. In both the analysed muscles, ASC-EVs treatment could only partially affect the maturation and innervation of NMJs with a mild increase of mature mono-innervated NMJs and a consequently reduction of immature multi-innervated and denervated NMJs.

Altogether, here we demonstrated that ASC-EVs induced beneficial effects in SMNΔ7 mice, model of a severe SMA, recapitulating the neuroprotective effects of their parental mesenchymal stem cells. Indeed, we confirmed their ability to counteract the disease progression, in particular the degeneration of MNs and the neuroinflammation in the lumbar spinal cord. ASC-EVs seem to be promising candidates for SMA therapy, possibly in combination with SMN-dependent drugs to boost their effects, even if the mechanisms of action involved remain to be better clarified and further studies could better explain the EVs homing and uptake mechanisms, after their administration. Indeed, it is crucial to understand EVs biodistribution in the target organs, to be able to predict their therapeutic response. Perets and co-workers recently developed a system to track the migration and homing of EVs derived from bone marrow MSCs in vivo in different brain diseases (including stroke, autism, Parkinson’s and Alzheimer’s diseases). They found that the accumulation of MSC-EVs correlates with inflammatory signals in pathological brains [76, 77] and this could be strictly related to the markers expressed on their surface, such as integrins [78].

Finally, the ICV injections used in this study was chosen to directly deliver ASC-EVs to the CNS; indeed, the passage of the blood-brain barrier and the delivery of therapeutic agents efficiently to the CNS represents one of the main issues in the treatment of neurodegenerative diseases. However, the chosen administration route remains controversial due to its invasiveness. To overcome this problem, the intranasal administration route represents a very attractive strategy as it is a non-invasive way, it avoids the hepatic metabolism and the systemic absorption and a multidose therapeutic regimen, with the assistance of simple devices (drops, aerosol, spray), becomes more accessible even for patients with neurodegenerative disorders [79, 80]. Several studies reported beneficial effects of EVs injected via the i.n. route in different models, as in a mouse model of Alzheimer’s disease [81], in the treatment of brain inflammatory diseases [82] and in our already published work using an in vivo ALS model [41]. Further efforts in this sense could be done also for SMA condition, in order to deliver ASC-EVs to the CNS in a tolerable and easy way, as well.

## Conclusions

In this study we observed that ASC-EVs treatment may exert a neuroprotective effect at the central level as well as in terms of rescue of motor performances when administered in SMNΔ7 mice, model of SMA type II. This could support the development of an ASC-EVs-based therapy as a valid alternative to the use of MSCs, since their comparable neuroprotective effects. Furthermore, the EVs-based approach could be a safer and more easily accessible option in the field of neurodegenerative diseases, as well as of other pathological conditions, thanks to their characteristics since it would give the possibility to avoid the stem cells-related ethical and technical challenges and limitations. Finally, regarding more specifically the SMA disease, ASC-EVs could also possibly be imagined as a synergic therapy in combination with SMN-dependent drugs to enhance their beneficial effects for the treatment of SMA patients.

### Electronic supplementary material

Below is the link to the electronic supplementary material.

Supplementary Material 1

Supplementary Material 2

Supplementary Material 3

## Data Availability

The datasets used and/or analysed during the current study are available from the corresponding author on reasonable request.

## Abbreviations

ALS: Amyotrophic lateral sclerosis
ASC: EVs-ASC-derived extracellular vesicles
ASCs: Adipose-derived mesenchymal stem cells
BCA: Bicinchoninic protein assay
BGTX: Bungarotoxin
BSA: Bovine serum albumin
DMEM: Dulbecco’s Modified Eagle Medium
EVs: Extracellular vesicles
FBS: Fetal Bovine Serum
FL: SMN-Full-length SMN protein
GFAP: Anti-glial fibrillary acidic protein
GM130: Golgi matrix protein 130
H/E: Hematoxylin-eosin staining
HRP: Horseradish Peroxidase
HSP70: Heat Shock Protein 70
IBA1: Ionized calcium binding adaptor molecule 1
ICV: Intracerebroventricular
IGF1: Insulin-like growth factor
MNs: Motor neurons
MSCs: Mesenchymal stem cells
NDS: Normal donkey serum
NF: Neurofilament
NMJs: Neuromuscular junctions
NTA: Nanoparticle Tracking Analysis
PBS: Phosphate-buffered saline
PFA: Paraformaldehyde
SMA: Spinal muscular atrophy
SMI32: Neurofilament H
SMN: Survival motor neuron
TEM: Transmission Electron Microscopy
TSG101: Tumor susceptibility gene 101 protein
WT: Wild Type
