# Correction: Administration of adipose-derived stem cells extracellular vesicles in a murine model of spinal muscular atrophy: effects of a new potential therapeutic strategy

**DOI:** 10.1186/s13287-024-03744-x

**Published:** 2024-04-29

**Authors:** Federica Virla, Ermanna Turano, Ilaria Scambi, Lorenzo Schiaffino, Marina Boido, Raffaella Mariotti

**Affiliations:** 1 Department of Neuroscience, Biomedicine and Movement Sciences, University of Verona, Verona, Italy; 2 Neuroscience Institute Cavalieri Ottolenghi, Department of Neuroscience “Rita Levi Montalcini”, University of Turin, Turin, Italy

**Stem Cell Research & Therapy (2024) 15:94**

10.1186/s13287-024-03693-5

The production team handling the original article mistakenly omitted equal contribution of the final two authors, Marina Boido and Raffaella Mariotti. This designation has since been restored in the online version.

