# Supplementary material for: Administration of adipose-derived stem cells extracellular vesicles in a murine model of spinal muscular atrophy: effects of a new potential therapeutic strategy
Source: Stem Cell Res Ther. 2024 Apr 1;15:94. doi: 10.1186/s13287-024-03693-5 (PMC10986013; doi:10.1186/s13287-024-03693-5)
Supplement: Supplementary file 1 — Supplementary Material 1 [file 13287_2024_3693_MOESM1_ESM.docx]

**Administration of adipose-derived stem cells extracellular vesicles in a murine model of Spinal Muscular Atrophy: effects of a new potential therapeutic strategy**

Virla Federica^1^, Turano Ermanna^1^, Scambi Ilaria^1^, Schiaffino Lorenzo^1^, Boido Marina^2#^_,_ Mariotti Raffaella^1^*^#^

*^1^Department of Neuroscience, Biomedicine and Movement Sciences, University of Verona, Verona, Italy. federica.virla@univr.it (F.V.); ilaria.scambi@univr.it (I.S.); ermanna.turano@univr.it (E.T.); lorenzo.schiaffino@univr.it (L.S.); raffaella.mariotti@univr.it (R.M.).*

*^2^Neuroscience Institute Cavalieri Ottolenghi, Department of Neuroscience "Rita Levi Montalcini", University of Turin, Turin, Italy. marina.boido@unito.it (M.B.).*

# These authors contributed equally to this work

*Corresponding author: raffaella.mariotti@univr.it


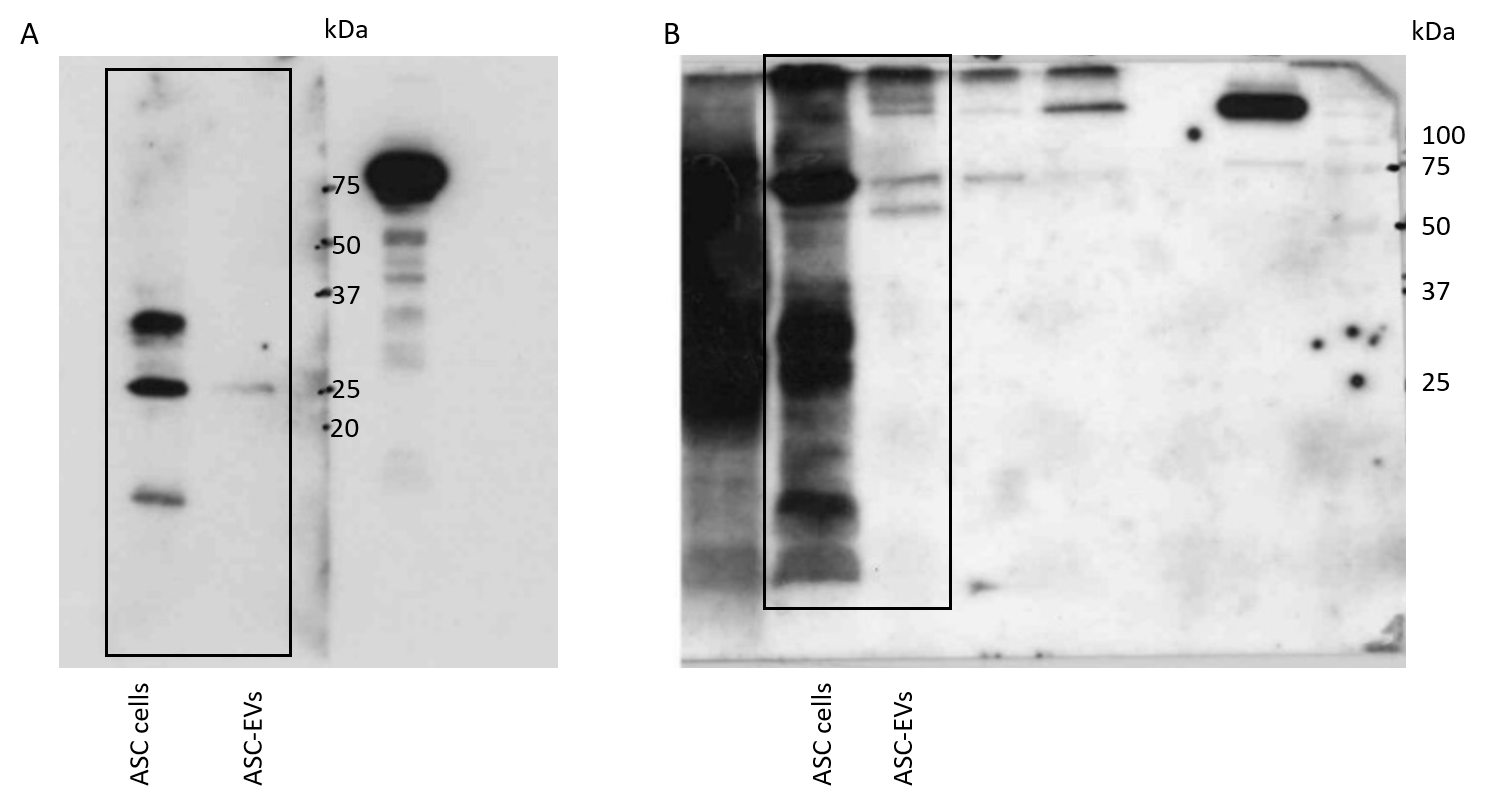


**Additional figure 1:** Western blot of specific EVs protein markers in ASCs lysates and ASC-EVs. The figure shows full-length western blots of gels presented in Figure 1c of the main article. In (a) CD9 and in (b) HSP70. The protein molecular weight marker (kDa) is indicated on the right.
