# Supplementary material for: Administration of adipose-derived stem cells extracellular vesicles in a murine model of spinal muscular atrophy: effects of a new potential therapeutic strategy
Source: Stem Cell Res Ther. 2024 Apr 1;15:94. doi: 10.1186/s13287-024-03693-5 (PMC10986013; doi:10.1186/s13287-024-03693-5)
Supplement: Supplementary file 2 — Supplementary Material 2 [file 13287_2024_3693_MOESM2_ESM.docx]

**Figure 1.** Characterization of ASC-EVs. (A) ASC-EVs size and concentration were measured and analysed by NTA. (B) Representative transmission electron microscopy images of ASC-EVs showed particles with characteristic morphology and size (scale bar 100 nm). (C) Western blot analysis of specific EVs protein markers: bands at 25, and 75 kDa were present after incubation with CD9 and HSP70 antibodies, respectively. ASCs lysates were used as positive control.

**Figure 2.** ASC-EVs treatment improves the motor performance of SMA mice. From P2/P4 to P10 the body weight measurements (A), tail suspension test (B), righting reflex test (C) and negative geotaxis test (D), were used to evaluate the motor performance of SMA mice treated with PBS (SMA-PBS, grey line) or ASC-EVs (SMA-EVs, blue line) and WT mice (black line). Overall, the results suggest an improvement in behavioural and motor performances of SMA-EVs mice compared to the SMA-PBS group.

Data are shown as mean±SEM and were analysed by Two-way ANOVA mixed-effects model with Geisser-Greenhouse correction followed by Sidak’s multiple comparison post hoc test. Statistical difference between the groups are indicated (*p<0.05; **p<0,005; ***p<0,0005; ****p<0.0001).

Legend: * = SMA-EVs vs. SMA-PBS; ° = SMA-EVs vs. WT; # = SMA-PBS vs. WT

**Figure 3.** Effect of ASC-EVs administration on lumbar MNs degeneration in SMA mice. (A) The graph shows the quantification of MNs density (MNs number/volume) in the ventral horns of L1-L5 spinal cord for SMA-PBS (white), SMA-EVs (grey) and WT (black) group at P10. The treatment with ASC-EVs significantly protect MNs from neurodegeneration compared to control mice (One-way ANOVA ***p<0.0010; ****p<0.0001). (B) Representative Nissl stained sections of lumbar spinal cord of SMA-PBS, SMA-EVs and WT mice. The arrows show stained MNs. Scale bar 500 μm. (C) The graph shows the quantification of the percentage of MNs (SMI32+ cells) expressing Casp3 in SMA-PBS (white), SMA-EVs (grey) and WT (black) groups: the treatment with ASC-EVs decreased the activation of apoptotic marker Casp3 compared to control mice (One-way ANOVA **p=0.0050). (D) Representative confocal images showing Casp3+ (red) and SMI32+ (green) cells in the ventral horns of PBS- and ASC-EVs treated SMA and WT mice. Cell nuclei are labelled by DAPI staining (blue). The arrows show Casp3+/SMI32+ cells. Scale bar 50 μm.

**Figure 4.** ASC-EVs treatment modulates the neuroinflammation in SMA mice. (A) The graph shows the quantification of the percentage of astrogliosis (GFAP+ signal) in the ventral horns of L1-L5 spinal cord for SMA-PBS (white), SMA-EVs (grey) and WT (black) mice at P10. The ASC-EVs administration significantly decreased the percentage of GFAP-immunopositive profiles in SMA-EVs mice compared to SMA-PBS ones (One-way ANOVA **p=0.0040; ****p<0,0001). (B) Representative confocal images showing GFAP+ (red) cells in the ventral horns of PBS- (SMA-PBS) and ASC-EVs treated (SMA-EVs) SMA mice and WT mice. Cell nuclei are labelled by DAPI staining (blue). Scale bar 50 μm. (C) The graph shows the quantification of the percentage of microglial cells (IBA-1+ signal) in the ventral horns of L1-L5 spinal cord for SMA-PBS (white), SMA-EVs (grey) and WT (black) mice at P10. No differences in the percentage of IBA-1 immunopositive profile were observed between SMA-PBS, SMA-EVs and WT mice (One-way ANOVA p>0.05). (D) Representative confocal images showing IBA-1+ (red) cells in the ventral horns of PBS- (SMA-PBS) and ASC-EVs treated (SMA-EVs) SMA mice and WT mice. Cell nuclei are labelled by DAPI staining (blue). Scale bar 50 μm. (E) The graph displays the microglial cell classification in ramified, bushy or amoeboid, based on their shape; the results are expressed as a percentage on the total number of IBA-1-positive cells for SMA-PBS (white), SMA-EVs (grey) and WT (black) group (Two-way ANOVA *p<0.05; **p<0.0050). (F) Representative confocal images showing IBA-1+ (red) microglial cells “ramified”, “bushy” and “amoeboid” in the ventral horns of spinal cord. Cell nuclei are labelled by DAPI staining (blue). Scale bar 50 μm.

**Figure 5.** Effect of ASC-EVs treatment on skeletal muscles of SMA mice. The graphs show the quantitative analysis of the mean fiber area and Feret’s max diameter of gastrocnemius muscle (A) and quadriceps muscle (B) for SMA mice treated with PBS (SMA-PBS, white) or with ASC-EVs (SMA-EVs, grey) and for WT mice (black). ASC-EVs treatment can partially rescue the atrophy of skeletal muscle fibers in SMA mice. (One-way ANOVA *p<0.05; **p<0,005; ***p<0,0005). (C) Hematoxylin/eosin (H/E) stained representative images showing SMA-PBS, SMA-EVs and WT quadriceps fibers. Scale bar 50 μm. (D) Representative confocal image showing NMJs (αBGTX, red; NF-H, green, DAPI blue) in quadriceps muscle of SMA-PBS group of SMA-PBS. The arrows show three different NMJs phenotypes: mono-innervated (a single NF is contacting the endplate), multi-innervated (several NFs are contacting the endplate) and denervated (no co-localization between NF-H and αBGTX). Scale bar 25 μm. The graphs show the quantification of the percentage of mono-innervated, multi-innervated and denervated NMJs in gastrocnemius muscle (E) and quadriceps muscle (F) in SMA mice treated with PBS (SMA-PBS, white) or with ASC-EVs (SMA-EVs, grey) and in WT mice (black). Statistical difference between the groups are indicated (Two-way ANOVA *p<0.05).

**Suppl. Figure 1.** Evaluation of the invasiveness of the surgery in a WT animal. The image shows a section of P10 WT brain after ICV administration of 2 μl of ASC-EVs at P3 and P6: the brain tissue does not display any lesions in the areas interested by the injections. Scale bar 500 μm.
