# Supplementary material for: Administration of adipose-derived stem cells extracellular vesicles in a murine model of spinal muscular atrophy: effects of a new potential therapeutic strategy
Source: Stem Cell Res Ther. 2024 Apr 1;15:94. doi: 10.1186/s13287-024-03693-5 (PMC10986013; doi:10.1186/s13287-024-03693-5)
Supplement: Supplementary file 3 — Supplementary Material 3 [file 13287_2024_3693_MOESM3_ESM.docx]

ADDITIONAL FILES

**
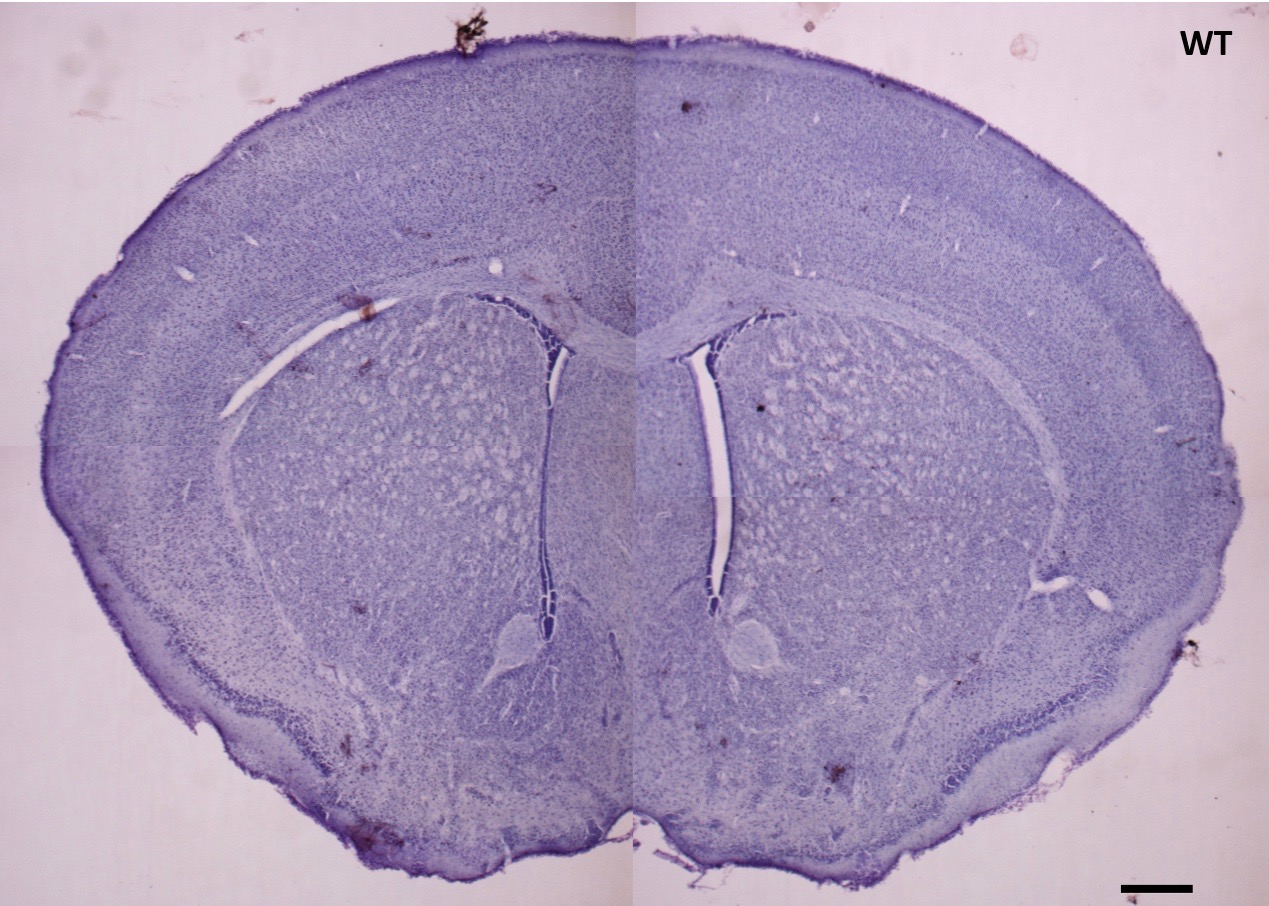
**

**Suppl. Figure 1.** Evaluation of the invasiveness of the surgery in a WT animal. The image shows a section of P10 WT brain after ICV administration of 2 μl of ASC-EVs at P3 and P6: the brain tissue does not display any lesions in the areas interested by the injections. Scale bar 500 μm.
